# In-season weather data provide reliable yield estimates of maize and soybean in the US central Corn Belt

**DOI:** 10.1007/s00484-020-02039-z

**Published:** 2020-11-21

**Authors:** Vijaya R. Joshi, Maciej J. Kazula, Jeffrey A. Coulter, Seth L. Naeve, Axel Garcia y Garcia

**Affiliations:** 1 Department of Agronomy and Plant Genetics, University of Minnesota, St. Paul, MN 55108 USA; 2 Southwest Research and Outreach Center, University of Minnesota, 23669 130th Street, Lamberton, MN 56152-1326 USA; 3 Department of Agricultural and Biological Engineering, University of Florida, Gainesville, FL 32611 USA

**Keywords:** Yield forecasting, Statistical modeling, Weather index, Crop modeling

## Abstract

Weather conditions regulate the growth and yield of crops, especially in rain-fed agricultural systems. This study evaluated the use and relative importance of readily available weather data to develop yield estimation models for maize and soybean in the US central Corn Belt. Total rainfall (Rain), average air temperature (Tavg), and the difference between maximum and minimum air temperature (Tdiff) at weekly, biweekly, and monthly timescales from May to August were used to estimate county-level maize and soybean grain yields for Iowa, Illinois, Indiana, and Minnesota. Step-wise multiple linear regression (MLR), general additive (GAM), and support vector machine (SVM) models were trained with Rain, Tavg, and with/without Tdiff. For the total study area and at individual state level, SVM outperformed other models at all temporal levels for both maize and soybean. For maize, Tavg and Tdiff during July and August, and Rain during June and July, were relatively more important whereas for soybean, Tavg in June and Tdiff and Rain during August were more important. The SVM model with weekly Rain and Tavg estimated the overall maize yield with a root mean square error (RMSE) of 591 kg ha^−1^ (4.9% *n*RMSE) and soybean yield with a RMSE of 205 kg ha^−1^ (5.5% *n*RMSE). Inclusion of Tdiff in the model considerably improved yield estimation for both crops; however, the magnitude of improvement varied with the model and temporal level of weather data. This study shows the relative importance of weather variables and reliable yield estimation of maize and soybean from readily available weather data to develop a decision support tool in the US central Corn Belt.

**Supplementary Information:**

The online version contains supplementary material available at 10.1007/s00484-020-02039-z.

## Introduction

Maize (*Zea mays* L.) and soybean [*Glycine max* (L.) Merr.] production in the US central Corn Belt is predominantly rain fed and highly dependent on in-season weather conditions (Franzluebbers et al. 2011; Green et al. 2018). In several areas of this region, weather variability has been reported to account for more than 75% of maize yield variation (Ray et al. 2015). Across the USA, 40 and 37% yield variability in maize and soybean was explained by in-season weather conditions, respectively, with an even greater percentage in the Corn Belt (Leng et al. 2016). Precipitation and air temperature during the growing season, particularly at late vegetative and early reproductive stages of development, are reported to cause significant yield deviations from average in both maize and soybean (Teasdale and Cavigelli 2017). Given the strong dependency of rain-fed maize and soybean yield on weather conditions, readily available meteorological data, such as average air temperature (Tavg) and rainfall (Rain), can be used to develop yield estimation models as decision support tools. Yield estimation is one of the most important factors for decision-making on crop insurance, crop management, storage needs, and marketing (Peng et al. 2018). Thus, the development of a yield estimation model based on easily accessible weather data can aid farmers in making informed decisions (Chen et al. 2019) and policy-makers in drafting policies on trade and food security (Basso et al. 2013; van der Velde et al. 2019).

Several attempts have been made at developing weather-based crop yield estimation models. For example, Kaul et al. (2005) used field-specific weekly rainfall and United States Department of Agriculture (USDA) soil rating values to estimate maize and soybean yield at multiple locations in Maryland, USA. Using Tavg and Rain of June, July, and August with mid-May planting progress, Westcott and Jewison (2013) developed yield models for maize and soybean in the USA. The effects of delayed planting from unfavorable weather conditions at the beginning of the growing season were accounted in their model. However, mid-May planting progress, as measured by the percent of maize plantings by mid-May, can vary within a county and its effect can be difficult to estimate. In addition, the June Rain in their model was used as the deviation amount from normal and only in the years when the June Rain was lower than 10% tail of its distribution. Data on such weather variable are not always easily available and requires calculation before it can be used in the yield estimation model. Mathieu and Aires (2016, 2018) compared more than 50 agro-climatic indices and different weather-impact models for maize yield estimation in the USA. They found that the Standardized Precipitation Evapotranspiration Index (SPEI) and Tavg in July were the best predictors. Derivation of agro-climatic indices such as SPEI, however, requires data of several other parameters, e.g., potential evapotranspiration, which may not be readily available. Apart from yield estimation, weather-based yield models have also been used to assess crop yield sensitivity to weather variation (Cai et al. 2014; Mourtzinis et al. 2015) and to predict yield in future or altered climatic conditions (D’Agostino and Schlenker 2016; Verón et al. 2015).

Weather-based modeling is one of several methods for crop yield estimation. Broadly, this method can be considered a statistical approach for yield estimation, in which empirical regression equations are developed between yield and other predictors such as several weather parameters collected across multiple site-years (Mathieu and Aires 2016; Thompson 1969). In weather-based models, weather data such as Tavg, Rain, or agroclimatic indices (e.g., Palmer drought severity index, SPEI, and growing degree days) are used as predictors in regression equations. In recent decades, with the advancement of remote sensing tools, several spectral indices such as normalized difference vegetation index (Kriegler et al. 1969; Rouse et al. 1974) and enhanced vegetation index (Huete et al. 2002) are also being used to develop crop yield estimation models (Franch et al. 2019; Prasad et al. 2006). Other approaches for crop yield estimation include field observation/sampling and crop simulation modeling. The field observation/sampling is based on careful observation or destructive measurements of representative crop samples. For example, the USDA estimates in-season crop yield based on grower-reported surveys and field-measurement surveys. These surveys are designed to collect data on cultivated area, crop density, row spacing, and yield components (USDA-NASS 2012). In crop simulation modeling, computer-based models are run with soil, weather, cultivar, and crop management information in order to estimate crop yield (Lobell and Asseng 2017; Morell et al. 2016). Crop models ranging in complexity and data requirements have been used in scientific research and as decision support tools (Jame and Cutforth 1996; Jones 1993; Setiyono et al. 2011).

Weather-based yield estimation models have several advantages over field observation/sampling and crop simulation modeling approaches. Field observation/sampling often entails trained personnel to collect representative samples from the field for meticulous measurements (USDA-NASS 2012). Therefore, the field observation/sampling approach can be time consuming and expensive. While crop modeling can be advantageous for estimating crop yield from the sub-field to national level (Fraisse et al. 2001; Morell et al. 2016; van Wart et al. 2013), it requires several soil and crop parameters for calibration and evaluation (Mathieu and Aires 2016; Verón et al. 2015). In contrast, weather-based models rely only on weather parameters or comparatively fewer parameters. Therefore, these models can be advantageous in terms of cost and scalability in both temporal and spatial domains. In previous studies, weather-based models have been used in yield estimation ranging from short term, such as the current year, to long term, such as at the mid or end of the century under future climate change scenarios (Chen et al. 2019; Schlenker and Roberts 2009). Similarly, regarding spatial scale, these models have been applied at the field, regional, and global levels (Lobell et al. 2008; Powell and Reinhard 2016; Ray et al. 2015). Despite these comparative advantages, weather-based models have some limitations. One of these arises from the nature of space-time data, which are often spatially and temporally correlated (Mathieu and Aires 2016; Verón et al. 2015). In addition, collinearity and interaction among the weather variables can make such data difficult to model (Shi et al. 2013). Moreover, the spatial and temporal levels of data used to develop a model can strongly influence the accuracy of yield estimation (Lobell and Burke 2010; Mathieu and Aires 2016). Even with these limitations, weather-based models or statistical models in general, are still widely used by the scientific community (Lobell and Burke 2010; Mathieu and Aires 2016; Shi et al. 2013).

For model development, multiple linear regression has been widely used, but is gradually being replaced by other more advanced approaches such as mixed-effects models (Mathieu and Aires 2016) and general additive models (Chen et al. 2019). Recent studies have also explored the potential of machine learning approaches, such as random forest model (Jeong et al. 2016), support vector machine regression (Oguntunde et al. 2018), and neural network (Crane-Droesch 2018). As the availability of weather data has increased with improvement in temporal frequency and spatial resolution of data, more advanced statistical models provide the opportunity to develop more accurate crop yield estimation models. Most studies have used Rain, Tavg, solar radiation, and vapor pressure on monthly or seasonal levels as predictors (Lobell et al. 2014; Mathieu and Aires 2016; Schlenker and Roberts 2009; Tack et al. 2015; Verón et al. 2015). A limited number of studies have used weather data at daily, weekly, and biweekly timescales and with more advanced statistical models for crop yield estimation (Kaul et al. 2005; Tack et al. 2015). While the difference between daily maximum and minimum air temperature (Tdiff) has been shown to significantly affect crop yield, its use in yield estimation models is less frequent (Hu et al. 2003; Lobell 2007; Tack et al. 2015; Verón et al. 2015). With relatively greater increment in daily minimum air temperature as compared to maximum air temperature, studies have shown a decrease in Tdiff on a global scale in the last 50 years (Lewis et al. 2013; Vose et al. 2005). However, the role of Tdiff in maize and soybean yield estimation model development is poorly understood. Understanding the implication of Tdiff on maize and soybean production is crucial for designing resilient cropping systems in the US Corn Belt and global food security, in general. The overarching goal of this study is to assess yield estimation models for maize and soybean grown in the US central Corn Belt based on the hypothesis that in rain-fed agricultural systems of the region, in-season weather data can be used to estimate maize and soybean yield. The specific objectives are to (1) compare the accuracy of multiple linear regression (MLR), general additive model (GAM), and support vector machine (SVM) regression model to estimate maize and soybean yields and (2) evaluate if inclusion of Tdiff in the model improves model performance.

## Materials and methods

### Study period and study area

**Fig. 1:**
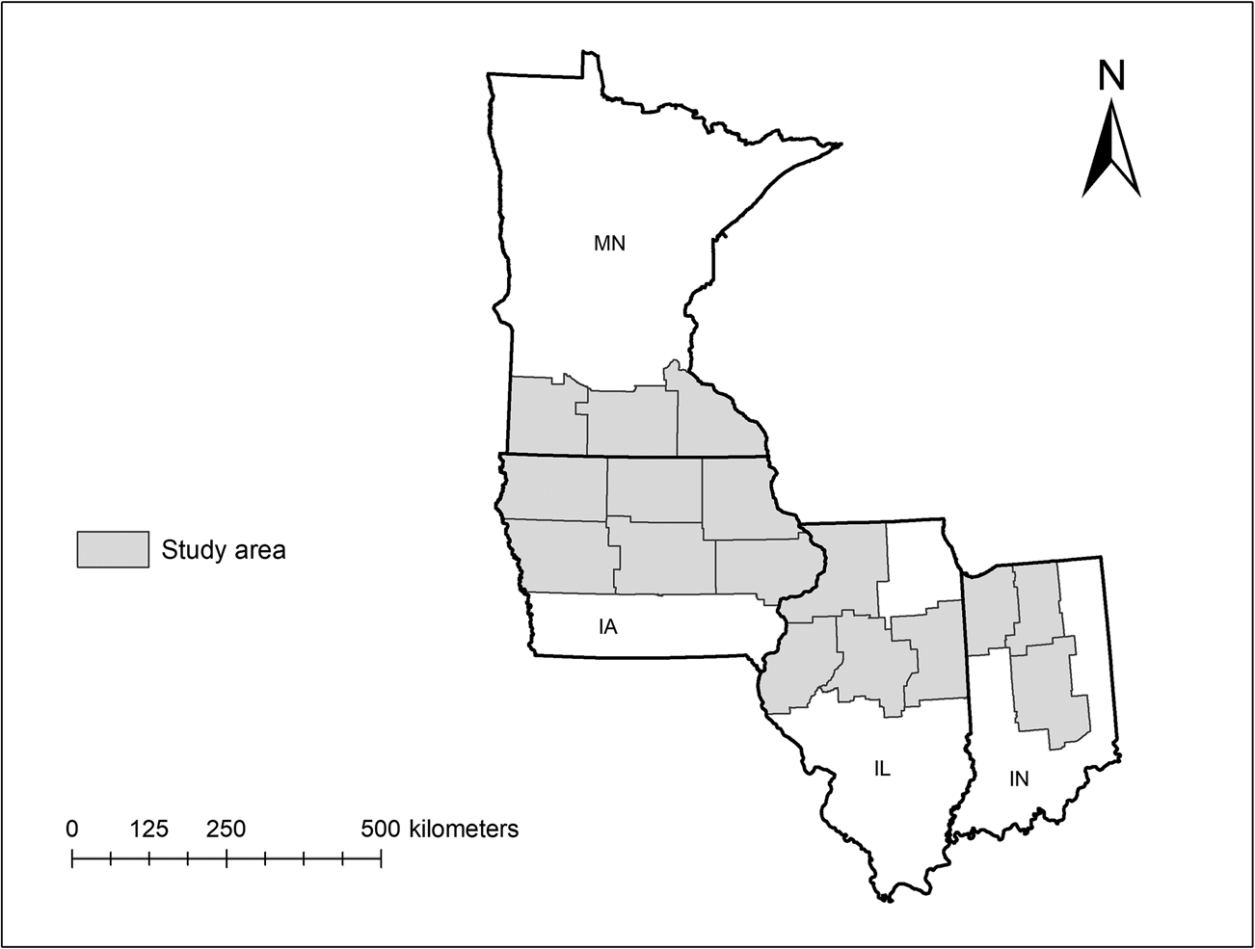
Map of the US states and the agricultural districts included in this study. In the map, MN, IA, IL, and IN refer to Minnesota, Iowa, Illinois, and Indiana, respectively

Historical weather data and grain yield of maize and soybean for a period of 28 years (1990–2017) were included for this study. The study year was selected until 2017 since the daily weather data at county level were available until 2017 at the time of data analysis. The study was limited up to 1990 only as it allowed considering uniform effects from the genetic, agronomic, and technological advancements in crop yield over the study period. The study area focused on the central Corn Belt region of the USA and included the major rain-fed maize and soybean agricultural districts of Iowa (IA), Illinois (IL), Indiana (IN), and Minnesota (MN). These four states accounted for almost 50 and 44% of total US maize and soybean grain production in 2018 (USDA-NASS 2019). The western Corn Belt region has a more irrigated production system and, therefore, was not considered for this study. From IA, IL, IN, and MN, only those agricultural districts with non-irrigated maize and soybean production were included. The number of counties included from IA, IL, IN, and MN were 68, 39, 33, and 29, respectively (Fig. 1). Altogether, the study involved a 28-year period and 169 counties from 16 agricultural districts in four states comprising a total data set of 4732 site-years.

### Data collection

Daily data on Tavg and Rain were obtained at the county level from the fine-scaled weather data set for contiguous USA developed by Schlenker and Roberts (2009) and Schlenker (2018) based on the PRISM Climate Group (2018) weather data. From the difference between daily maximum and minimum air temperatures, Tdiff was calculated. The data were prepared at weekly, biweekly, and monthly timescales.

County-level annual grain yield data of maize and soybean for the study period were obtained from USDA-NASS (2019) using “nass_data” function from “nassR” package (Dinterman and Eyer 2018) in R (R Core Team 2018).

### Model development

Yield estimation models were developed for each crop at the total study area level and at individual state level. For model development, in-season (May–August) weather data of Tavg and Rain with/without Tdiff were used as weather variables. Three different statistical models were assessed, which included MLR, GAM, and SVM. Each model was developed with weather data at weekly, biweekly, and monthly timescales.

First, time-series yield data were linearly detrended for each of the 169 counties to remove non-weather effects such as advancement in technology, improved agronomic practices, and cultivar changes over years. In this study, we used US maize and soybean production data from 1990 to 2017, a relatively short period during which yield increase was largely linear, so the detrending approach used was linear. Linear detrending is a common approach for trend removal in crop yield-climate relationship studies, especially for the removal of yield increment in a shorter time period. Other frequently used detrending approaches are first differencing and non-linear regressions. After trend removal, MLR, GAM, and SVM yield estimation models were developed.

In MLR, the mean response variable, *y*, was modeled as a linear function of *n* explanatory variables *x*_1_, *x*_2_, *x*_3_, *x*_*n*_, and can be written as

1 $$ y={b}_0+{b}_1{x}_1+{b}_2{x}_2+{b}_3{x}_3+\cdots +{b}_n{x}_n+\epsilon $$

where *b* refers to a regression coefficient and *ϵ* is the residual. The regression coefficient in MLR is estimated based on the ordinary least square regression that minimizes the sum of squared errors. For this study, MLR was modeled as

2 $$ {y}_{i,j}={b}_0+\sum {\left(\beta .X\right)}_{i,j}+{\epsilon}_{i,j} $$

where *y*_*i,j*_ denotes the estimated yield of the *i*th county in the *j*th year, *b*_0_ refers to the intercept or mean yield, and *β* denotes a vector of regression coefficients for *X* vector of weather variables, namely Tavg, Rain, and Tdiff from May to August.

In GAM, the linear relationship as described in MLR was modeled as a smooth function in order to capture any non-linearities in the model (Hastie and Tibshirani 1990), and can be written as

3 $$ y={b}_0+{f}_1{x}_1+{f}_2{x}_2+{f}_3{x}_3+\cdots +{f}_n{x}_n+\epsilon $$

where *f* refers to smooth functions on explanatory variable *x*, which is estimated using a scatterplot smoother (Hastie and Tibshirani 1990). For this study, GAM was modeled as

4 $$ {y}_{i,j}={b}_0+\sum \kern0.5em {(F.X)}_{i,j}+{\epsilon}_{i,j} $$

where *F* denotes a vector of smoother functions for *X* vector of weather variables as mentioned above.

In SVM, a regression function is set up from a training dataset with a margin of tolerance defined by *ϵ* as the *ϵ*-insensitive zone. The SVM ignores any error from data points within this margin of tolerance. Non-negative slack variables outside this margin are used to measure the deviation of training samples. The goal is to find an optimal hyperplane by choosing the function that minimizes the deviation from the insensitivity parameter (Cortes and Vapnik 1995; Vapnik 1999). In a given dataset of (*x*_*i*_, *y*_*i*_) pairs, where *i* refers to 1,.., *n* observations, a linear function in SVM can be expressed as

5 $$ y=f(x)=\omega .x+b $$

where *ω*. *x* denotes the product between weighing vector *ω* and input vector *x*, and *b* refers to the bias term. The objective function in regression analysis using SVM is to minimize *ω*. Errors less than *ϵ* (tolerance margin) are not considered for the objective function, and only the non-negative slack variables across the tolerance margin or *ϵ*-insensitive zone are considered (Vapnik 1999). In non-linear regression, the data are first linearized through incorporation of kernel functions to apply linear functions. Some of the commonly used kernels include polynomial, radial basis function, and sigmoid. In this study, radial basis function kernel was used for SVM.

All data analyses were performed in R v. 3.5.1 (R Core Team 2018) using the caret package (Kuhn 2008). The datasets were randomly allocated for training (75% of total data) and testing (25% of total data) for model development and evaluation, respectively. For MLR and GAM, data were transformed using the BoxCox transformation (Box and Cox 1964), whereas for SVM, the data were centered and scaled before processing. Each model was trained using fivefold cross validation with five repetitions. For the MLR model, the “lmStepAIC” method was used for stepwise selection of variables and selection of the model with the lowest AIC value. For the GAM model, the “gam” method from the “mgcv” package (Wood 2018) was used. For the SVM model, the “svmRadial” method from the “kernlab” package (Karatzoglou et al. 2018) was used. The MLR did not have tuning parameters. For GAM, the smoothing parameter estimation method was set to “REML” and the selection type to “TRUE,” which penalizes for new terms in the model and allows removal of terms (Wood 2017, 2018). For SVM, the sigma and cost parameters were tuned using the “tuneLength” command, which was set to test 15 default values for each parameter.

### Variable importance

The relative importance of weather variables in estimating maize and soybean yields was determined using the “varImp” function in the caret package (Kuhn 2008) in R (R Core Team 2018). The relative importance was determined by using a locally weighted least square regression (loess) smoother between crop yield and each weather variable. The *R*^2^ value from each model was compared with the intercept-only null model, which was used to determine the relative importance of weather variables. Only the weather variables grouped at monthly scale were used for this purpose.

### Model evaluation

An independent dataset (not used for model development) was used for model evaluation. Model fitness was evaluated using the root mean square error (RMSE), normalized RMSE (*n*RMSE), correlation coefficient (*r*), and Willmott’s index of agreement (*d*) (Willmott 1981).

The RMSE between the estimated and actual yield was calculated using the following equation:

6 $$ RMSE=\sqrt{\frac{\sum_{i=1}^n\ {\left({E}_i-{A}_i\right)}^2}{n}} $$

where *n* refers to the number of observations, and *E* and *A* denote estimated and actual yield. Then, *n*RMSE was calculated as *RMSE*/mean(actual yield). Lower RMSE and *n*RMSE values indicate better model performance.

The *r* ranges from − 1 to + 1, with − 1 indicating perfect negative linear correlation and + 1 indicating perfect positive linear correlation between estimated and actual yields. It was calculated using the following equation:

7 $$ r=\frac{\sum_{i=1}^n\left({A}_i-\overline{A\Big)}\ \right({E}_i-\overline{E\Big)}}{\sqrt{\sum_{i=1}^n\Big({A}_i-{\overline{A\Big)}}^2\ }\sqrt{\sum_{i=1}^n\Big({E}_i-{\overline{E\Big)}}^2}} $$

The *d* varies between 0 and 1, with 1 indicating a perfect match and 0 indicating no match between estimated and actual yields and was calculated using the following equation:

8 $$ d=1-\frac{\sum_{i=1}^n\ {\left({A}_i-{E}_i\right)}^2}{\sum_{i=1}^n\ {\left(|\ {E}_i-\overline{\ A\ }\ |+|\ {A}_i-\overline{\ A\ }\ |\right)}^2}\kern0.5em $$

## Results

### Average maize and soybean yield and yield deviation

Over the total study area, the average yield for maize and soybean was 11,950 and 3680 kg ha^−1^, respectively (Table 1). The coefficient of variation (CV) was 12% for maize and 11.3% for soybean. For maize, the highest average yield was from MN, followed by IA, IL, and IN. The CV ranged from 9.1 to 13.3% across states. For soybean, the highest average yield was in IL, followed by IA. In IN and MN, the soybean yield was similar at around 3580 kg ha^−1^. The CV for soybean yield was 10.4–11.5% in all states.

**Table 1:** Total study area and state average detrended yield and coefficient of variation (CV) of maize and soybean during the study period (1990–2017)

State	Maize		Soybean	
	Average yield (kg ha^−1^)	CV (%)	Average yield (kg ha^−1^)	CV (%)
Iowa	12,260	9.8	3700	11.1
Illinois	12,160	12.2	3830	10.4
Indiana	10,740	13.3	3580	10.9
Minnesota	12,300	9.1	3580	11.5
Total area	11,950	12.0	3680	11.3

**Fig. 2:**
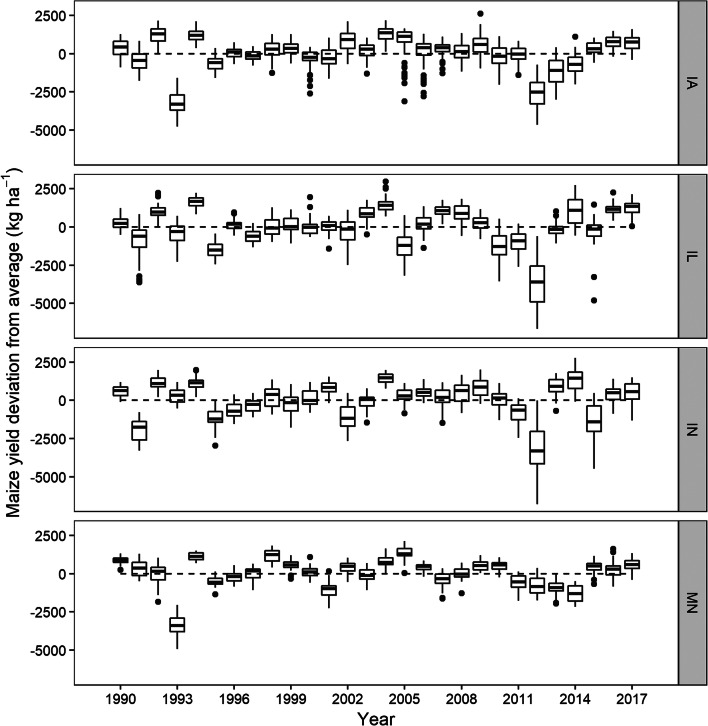
Maize yield deviation from average in Iowa (IA), Illinois (IL), Indiana (IN), and Minnesota (MN) from 1990 to 2017. The dotted black line represents the zero-reference line

**Fig. 3:**
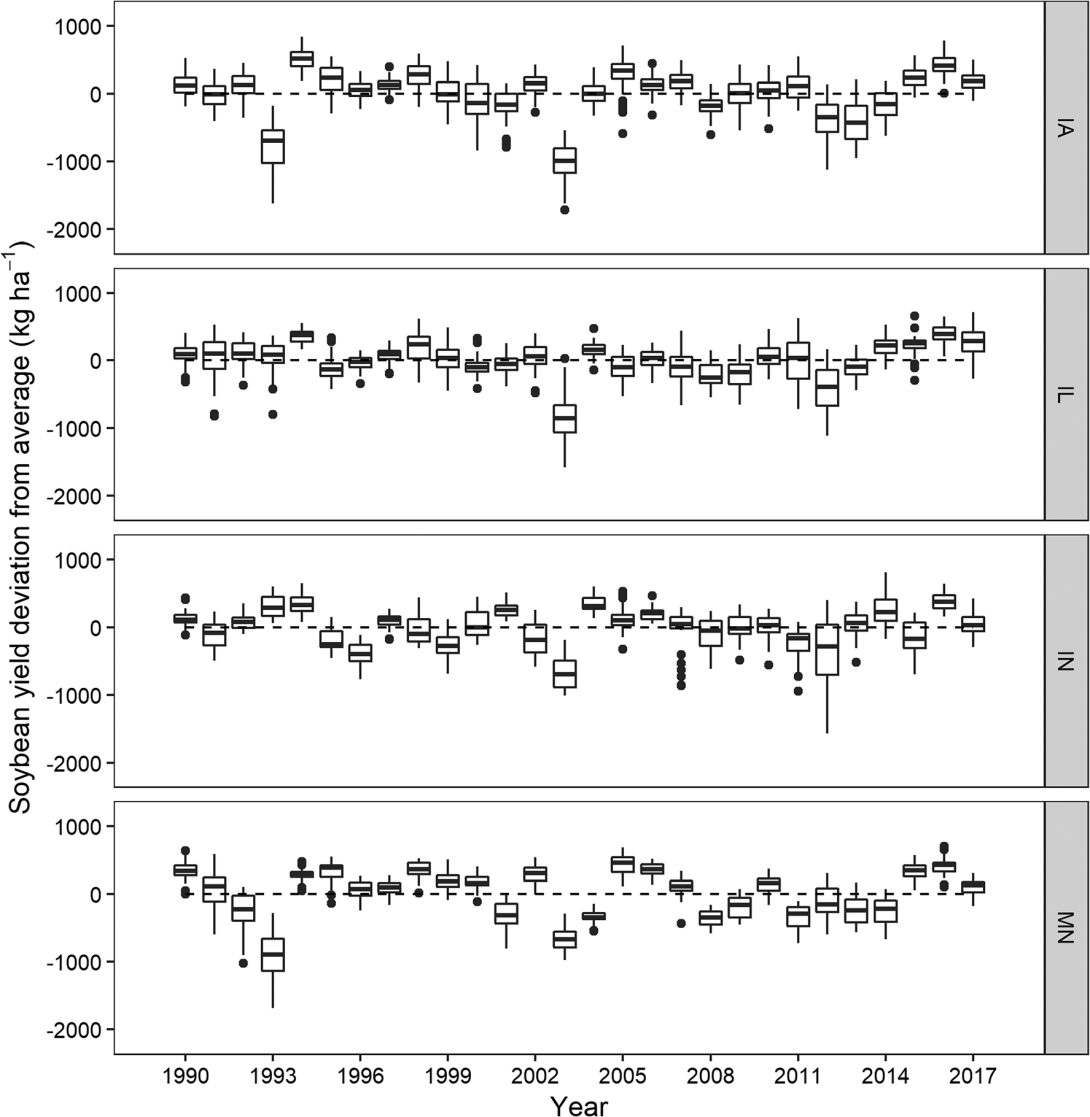
Soybean yield deviation from average in Iowa (IA), Illinois (IL), Indiana (IN), and Minnesota (MN) from 1990 to 2017. The dotted black line represents the zero-reference line

Considerable maize and soybean yield loss occurred in 1993, to a greater extent in IA and MN, due to flooding (Phillips 1994). Lower-than-average yield in soybean was observed in 2003, and can be attributed to dry conditions during August and September (Brumm and Hurburgh 2003). In all states, except MN, the highest negative maize and soybean yield deviation from average occurred in 2012, a historic drought year that caused a significant reduction in US crop yields (USDA-NASS 2013) (Figs. 2 and 3). In 1994, 2016, and 2017, comparatively, yield in all states was above average ranging from 293 to 1635 kg ha^−1^ in maize and from 49 to 508 kg ha^−1^ in soybean (Figs. 2 and 3).

### Model development

**Fig. 4:**
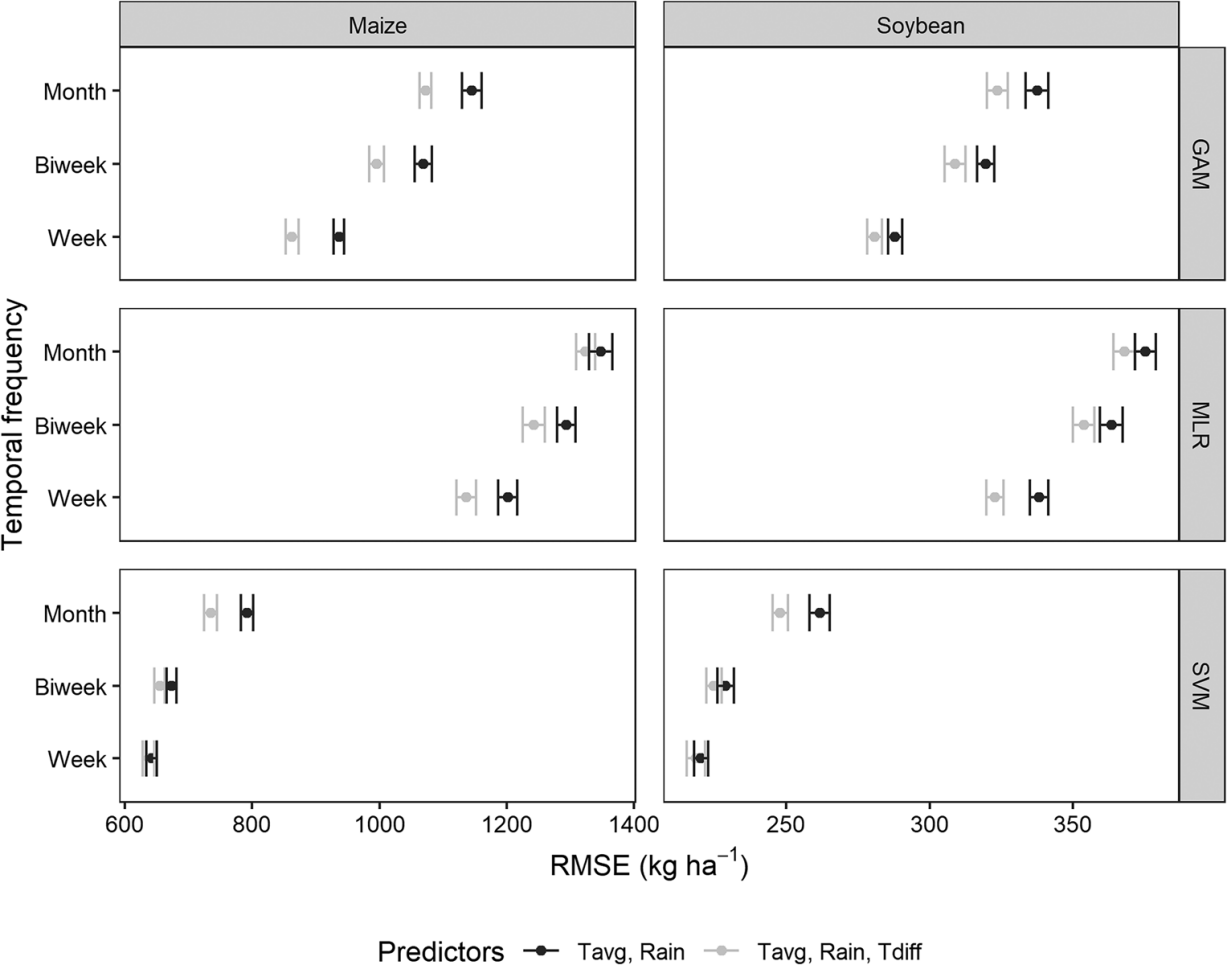
Root mean square error (RMSE) of maize and soybean yield estimation during model development for the generalized additive model (GAM), multiple linear regression (MLR), and support vector machine (SVM) models trained with weather predictors at weekly, biweekly, and monthly timescales

The SVM model predicted the maize and soybean yield with the lowest RMSE and therefore, performed the best followed by GAM and MLR at all temporal levels of weather data (Fig. 4). Inclusion of Tdiff in the model improved yield estimation for both crops; however, the magnitude of improvement varied with model and temporal level of weather data. With the SVM model for maize, inclusion of Tdiff at weekly level did not improve the yield estimation. Similarly, adding Tdiff in the MLR model did not improve yield estimation at monthly level. However, estimation improved significantly (*P* < 0.05) in all other cases. For soybean, inclusion of Tdiff in the SVM model did not improve the estimation until monthly level. Adding Tdiff to the GAM and MLR models significantly (*P* < 0.05) improved yield estimation at all levels.

Within SVM models for maize, no significant difference in yield estimation occurred between weekly and biweekly models; yet, the RMSE from both was significantly lower compared to the monthly model (Fig. 3). Similar results were obtained for soybean yield estimations. This reveals the greater efficiency and superior performance of the SVM model compared to the GAM and MLR models. At the state level, the SVM model produced the lowest RMSEs as shown in Figs. S1–S8 (Electronic Supplementary Material).

### Variable importance

**Fig. 5:**
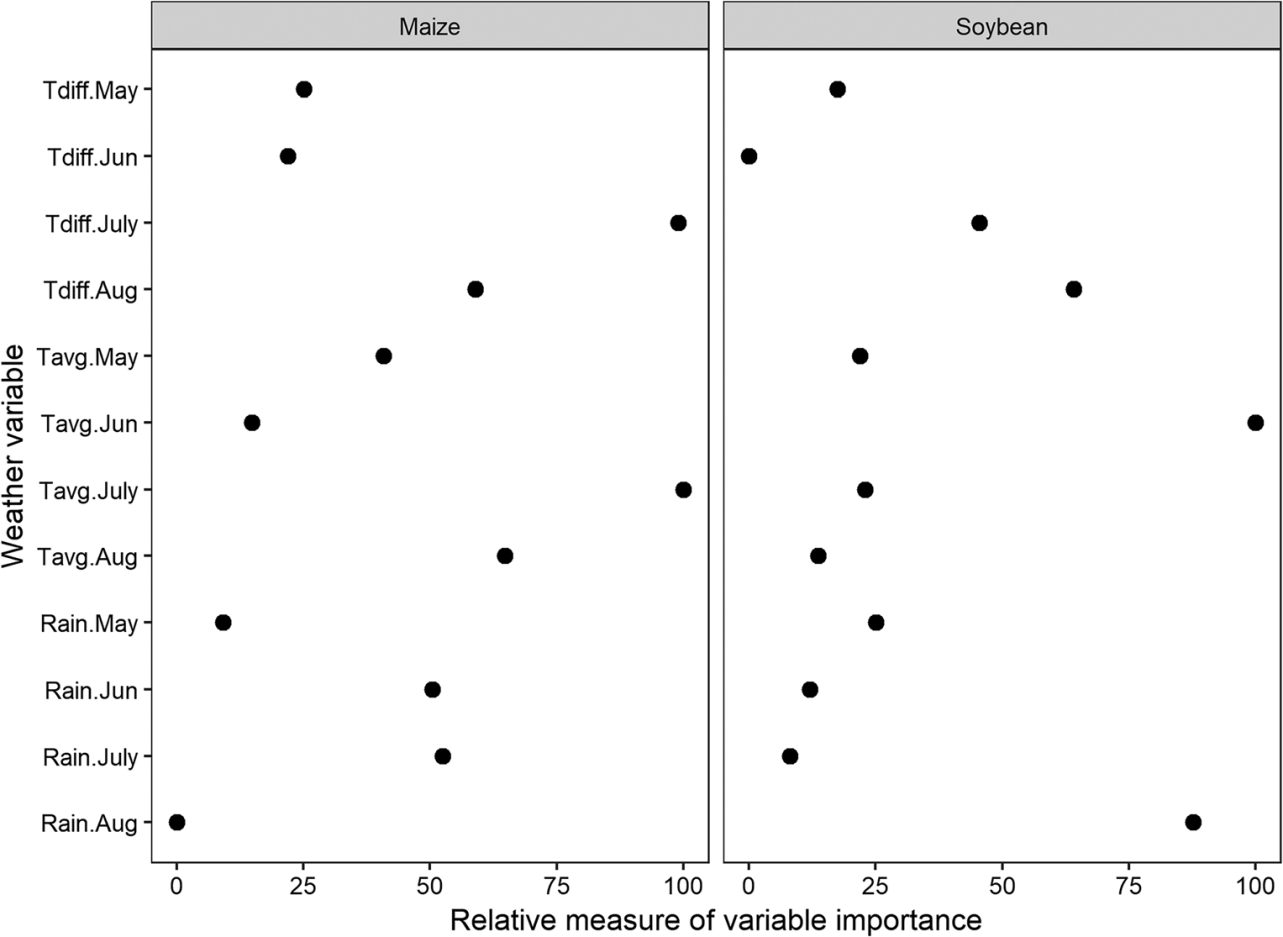
Relative measure of monthly weather variable importance scores in estimating maize and soybean yield across Iowa, Illinois, Indiana, and Minnesota. (Importance scores were normalized and scaled between 0 and 100)

In estimating maize yield across the study area, rainfall during June and July and temperature during July and August were more important than other monthly weather variables (Fig. 5). The Tavg and Tdiff during July and August were both important. Rainfall during May and August, which coincides with early vegetative and late reproductive stages in maize, respectively, were comparatively less important in estimating maize yield.

In estimating soybean yield, Tavg during June, when plants are in early reproductive stages, was the most important weather variable; rainfall during August was the second-most important variable (Fig. 5). As in maize, Tdiff during July and August was important for soybean yield estimation.

### Model evaluation

**Fig. 6:**
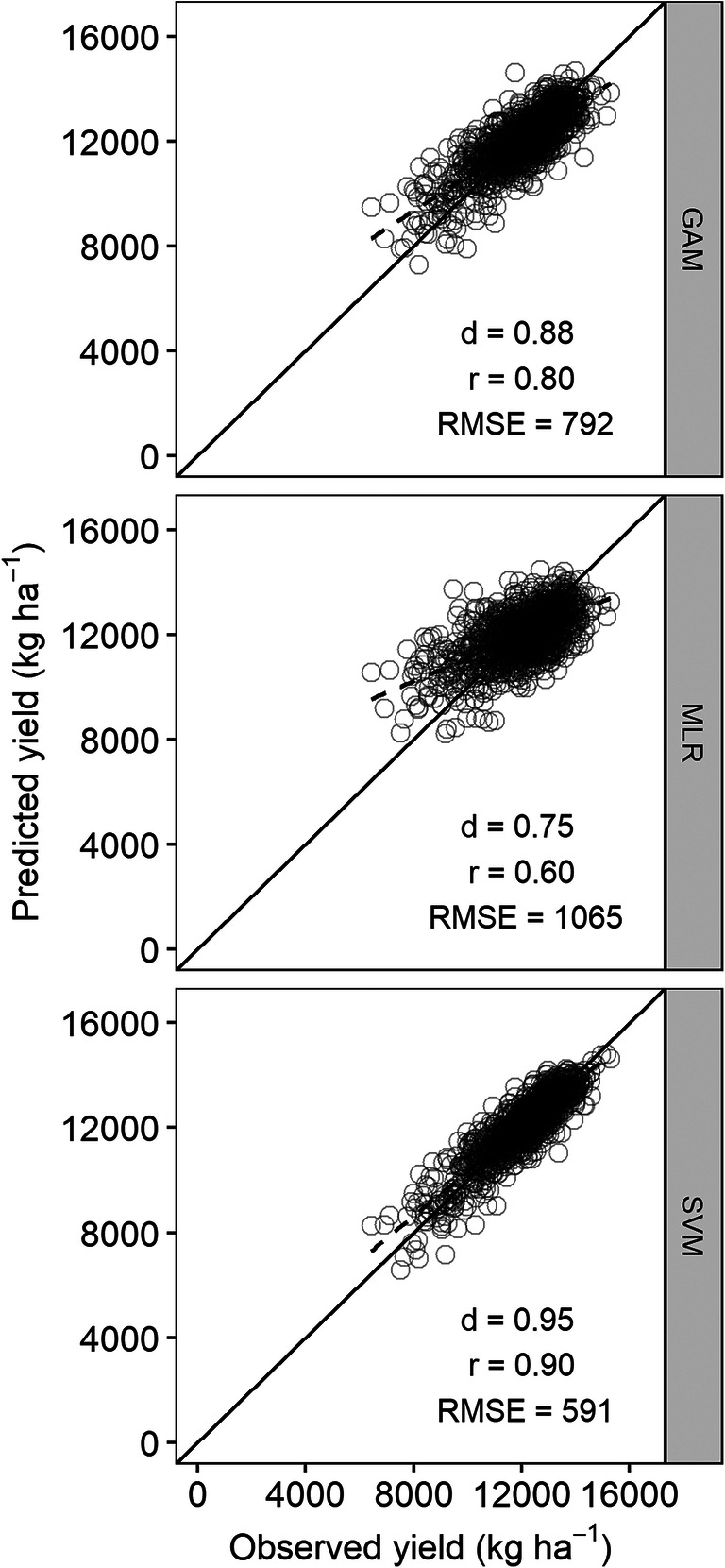
Scatterplots of observed versus predicted maize yield from the generalized additive model (GAM), multiple linear regression (MLR), and support vector machine (SVM) models. The diagonal black line is the 1:1 line. The dashed black line represents the linear regression between observed and predicted yields

The best-performing models based on the lowest RMSE (Fig. 4) were used to estimate maize and soybean yield using an independent test dataset that was not used previously for model development (Fig. 6). As in model development, the SVM model outperformed the other models. The SVM model with weekly weather data of only Tavg and Rain as predictors estimated maize yield with the lowest RMSE of 591 kg ha^−1^ (4.9% *n*RMSE). The *r* and *d*-index were also the highest for the SVM model. After the SVM model, the lowest RMSE was obtained from the GAM model (792 kg ha^−1^), followed by the MLR with RMSE of 1065 kg ha^−1^ (Fig. 6). The GAM and MLR models used weekly weather data of Tavg, Rain, and Tdiff.

**Fig. 7:**
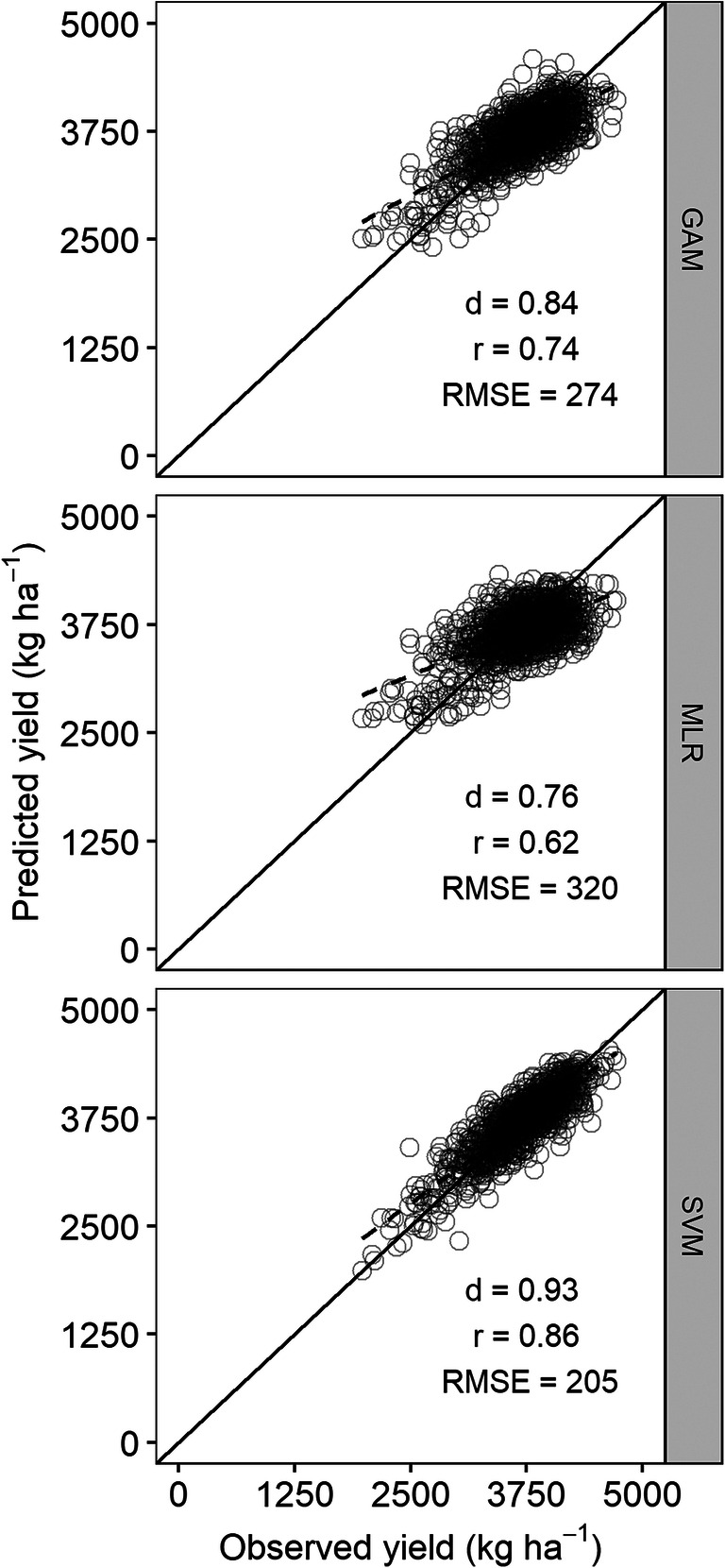
Scatterplots of observed versus predicted soybean yield from the generalized additive model (GAM), multiple linear regression (MLR), and support vector machine (SVM) models. The diagonal black line is the 1:1 line. The dashed black line represents the linear regression between observed and predicted yields

As in maize, the SVM model outperformed the other models in soybean yield estimation. The SVM model with weekly Tavg and Rain estimated soybean yield with the lowest RMSE of 205 kg ha^−1^ (5.5% *n*RMSE). Like in maize, the *r* and *d*-index were also highest for the SVM model. Similarly, the next lowest RMSE was obtained from the GAM model (274 kg ha^−1^) and the MLR model produced the highest RMSE of 320 kg ha^−1^ (Fig. 7).

### Yield estimation at state level

Models were also evaluated for both crops at individual state level. Since the SVM models gave the lowest RMSEs during model development (Figs. S1–S8, Electronic Supplementary Material), only those models were used for evaluation at the state level.

**Fig. 8:**
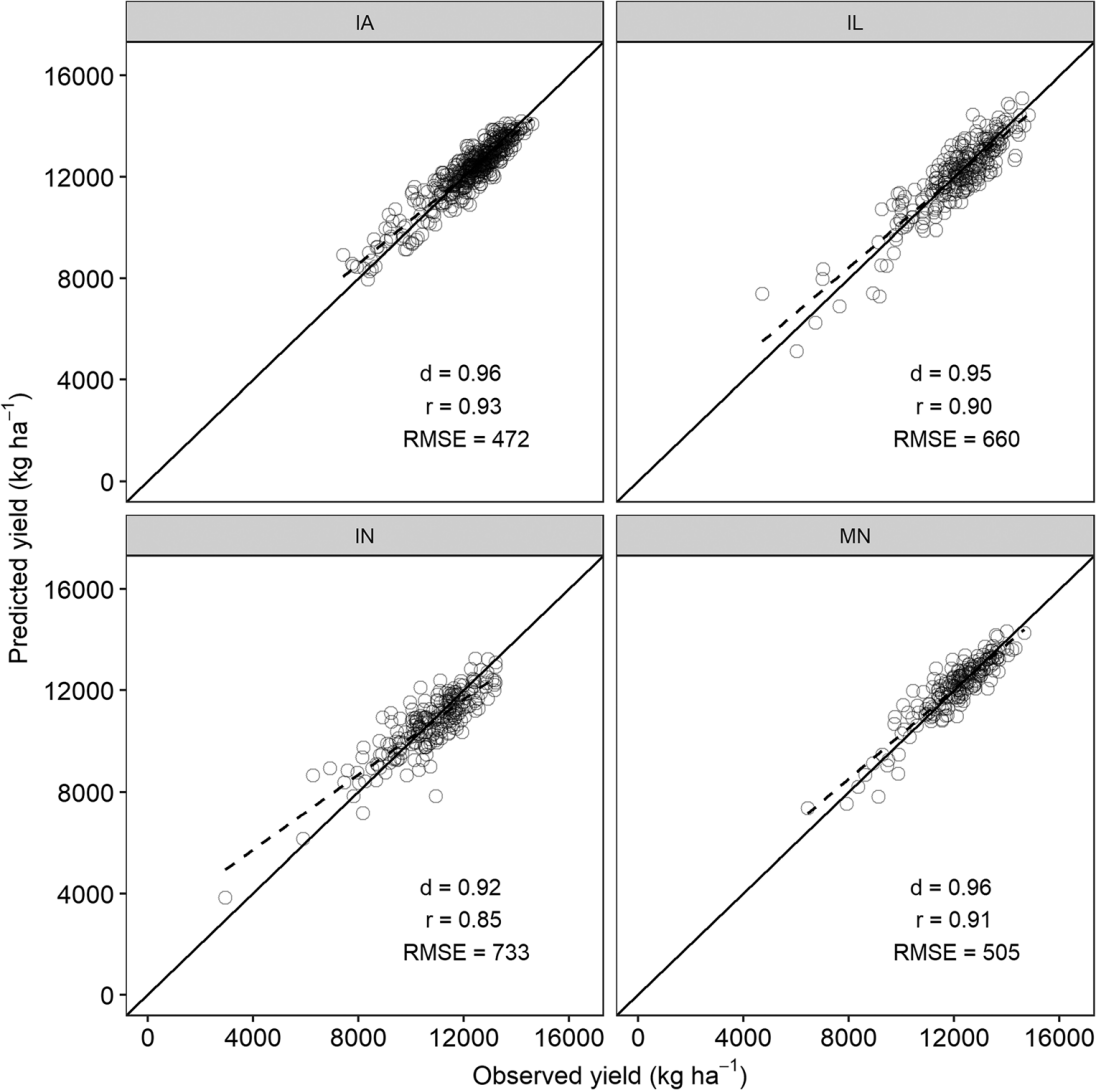
Scatterplots of observed versus predicted yield of maize using support vector machine model in Iowa (IA), Illinois (IL), Indiana (IN), and Minnesota (MN). The diagonal black line shows the 1:1 line. The dashed black line represents the linear regression between observed and predicted yields

Overall, maize yield was estimated with a RMSE lower than 800 kg ha^−1^ in all states (Fig. 8). The lowest RMSE of 472 kg ha^−1^ (3.8% *n*RMSE) was obtained for IA, followed by MN with 505 kg ha^−1^ (4.2% *n*RMSE), IL with 660 kg ha^−1^ (5.4% *n*RMSE), and IN with 733 kg ha^−1^ (6.8% *n*RMSE). The *d*-index values were above 0.9 in all states. Except for IN, the values for *r* were equal to or greater than 0.9 in other states.

**Fig. 9:**
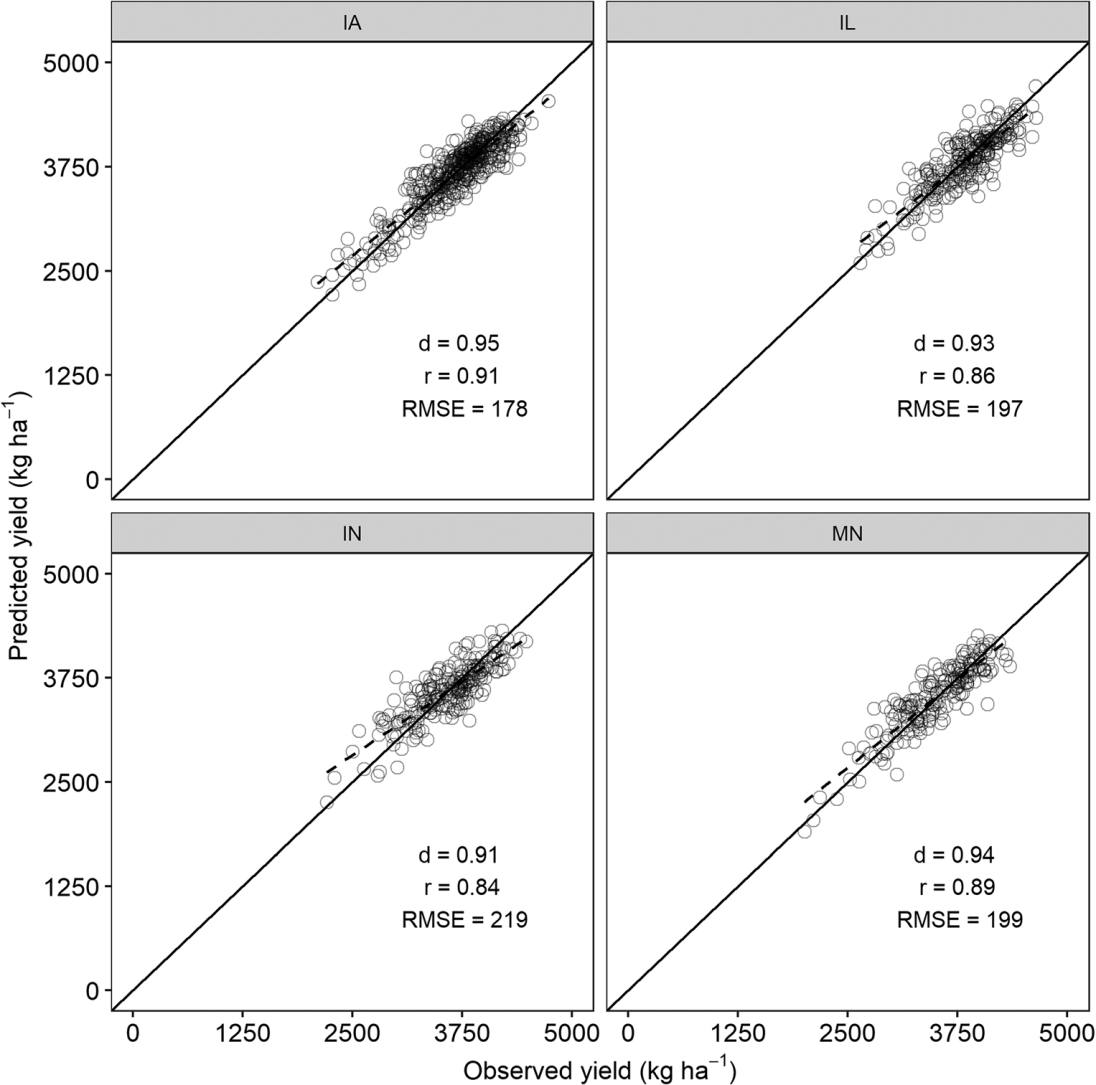
Scatterplots of observed versus predicted yield of soybean using support vector machine model in Iowa (IA), Illinois (IL), Indiana (IN), and Minnesota (MN). The diagonal black line shows the 1:1 line. The dashed black line represents the linear regression between observed and predicted yields

In all states, soybean yield was estimated with RMSE lower than 250 kg ha^−1^ (Fig. 9). The lowest RMSE for a soybean yield of 178 kg ha^−1^ (4.8% *n*RMSE) was obtained for IA, followed by IL with 197 kg ha^−1^ (5.1% *n*RMSE), MN with 199 kg ha^−1^ (5.7% *n*RMSE), and IN with 219 kg ha^−1^ (6.1% *n*RMSE). As for maize, the *d*-index values for soybean were also above 0.9 in all states. The values of *r* were 0.9, 0.86, 0.84, and 0.89 for IA, IL, IN, and MN respectively.

## Discussion and conclusions

Crop yield estimation is an important factor in the decision-making process for farm management. In this study, weather-based models for maize and soybean yield estimation were developed using easily available weather data of air temperature and rainfall. Weather variables show varied degree of correlation. For example, we found that average temperatures in June and July are positively correlated, whereas average air temperature and rainfall in June are negatively correlated (Fig. S9). Still, collinearity primarily influences the coefficients of a parameter and associated level of significance, but does not inhibit the predictive potential of the model (Neter et al. 1996). Additionally, covariance among weather variables in our training and test data is assumed to be similar as it is randomly allocated and large enough.

The SVM model clearly outperformed the GAM and MLR models. Using only Tavg and Rain data at the weekly level, the SVM model estimated county average maize yield with less than 5% *n*RMSE and soybean yield with less than 6% *n*RMSE (Figs. 6 and 7). Comparatively better yield prediction from the SVM than MLR has been reported in other studies as well. Chen et al. (2016) found that the SVM model was more accurate at predicting rice yield from weather variables than the MLR model. Similarly for maize, better model predictions with the SVM model compared to the MLR were reported by Karimi et al. (2008). Superior performance of the SVM model can be attributed to its ability to model nonlinear functions and high dimensional data (Vapnik 1999). Crop yield data in relation to weather parameters are nonlinear in nature (Lobell et al. 2011; Schlenker and Roberts 2009; Tack et al. 2015). Therefore, unsurprisingly, the MLR model which only modeled the linear relationship between weather variables and yield performed the worst in this study (Figs. 6 and 7). The GAM model, despite being able to model nonlinear relationships, did not give better estimations compared to the SVM model, but were better than those with the MLR model.

This study demonstrated the importance of Tdiff in maize and soybean yield estimation. Although the lowest RMSEs were obtained from models with Tdiff, the differences from models with and without Tdiff were not always significant (Fig. 4). Improvement in yield estimates with Tdiff were dependent on the temporal level of weather data, statistical model, and crop. Regardless of the temporal level of weather data, inclusion of Tdiff always significantly improved yield estimation with the GAM model (Fig. 4). Inclusion of Tdiff in the SVM model at the weekly level for maize and the weekly and biweekly levels for soybean did not improve yield estimates. However, including Tdiff in the model at the monthly level produced significantly lower RMSE for both crops. Yield estimates for maize and soybean obtained from monthly weather data with the SVM model were more accurate (lower RMSEs) than those from weekly and biweekly weather data from the GAM and MLR models.

The variable importance analysis showed that Tdiff played a key role in model development (Fig. 5). For maize, Tavg and Tdiff during July and August were important weather variables. Similarly, Tdiff during August and July were important for soybean. This highlights the important implication of Tdiff on maize and soybean yield. Lesser Tdiff owing to higher night temperature has been reported to reduce maize yield (Cantarero et al. 1999; Chang 1981; Peters et al. 1971) and soybean yield (Peters et al. 1971). Higher night temperature, especially during the reproductive phase, can reduce crop yield by increasing respiration and the duration of the reproductive phase (Cantarero et al. 1999; Chang 1981). Increased respiration reduces stored photo-assimilates in crops, thereby reducing their translocation to grains. Higher values of Tdiff have also been shown to reduce crop yields. Lobell (2007) found a nonlinear response of Tdiff to cereal grain yield and a negative response of maize yield to increased Tdiff. This finding can have an important implication on crop production and climate change studies. Most studies on climate change have focused primarily on the effects of increased air temperature, but the difference between maximum and minimum air temperatures has received less attention. Results from this study as well as similar results from past studies (Cantarero et al. 1999; Chang 1981; Lobell 2007) suggest that Tdiff is important to consider while assessing the impact of climate change on maize and soybean production. As the Tdiff has been decreasing on a global scale (Vose et al. 2005), it is critical to assess its impacts on future maize and soybean production in the US Corn Belt. A decrease in Tdiff due to warmer nights can be advantageous in some regions with too low air temperature as in northern MN but can be damaging to crops in regions with high daily air temperature near optimal. Understanding such implications of weather conditions on crop production will help to design cropping systems that are resilient to future weather situations.

This study demonstrates the usefulness of weather data for reliable estimation of maize and soybean yield in the US central Corn Belt. This study also highlights the comparative significance of weather variables in estimating maize and soybean yield. Improvements in weather-based crop yield estimation are highlighted with respect to different statistical models and weather variables. The comparative analyses of model performances indicated that the support vector machine model with radial kernel is better as compared to multiple linear regression and general additive model in estimating maize and soybean yields. The inclusion of the difference in daily maximum and minimum air temperature as an explanatory variable improved the performance of all three models assessed. However, such improvement was shown to be dependent on the temporal level of weather data, statistical model, and crop type. Due to inherent micro-environments in the agricultural landscape, future research should investigate the performance of such weather-based models in explaining spatial crop yield variability occurring within the field.

## Supplementary Information

ESM 1 (DOCX 11544 kb).

ESM 2 (CSV 24749 kb).

ESM 3 (CSV 24741 kb).

## Data Availability

All data analyzed during the study are included in this published article and its supplementary information files.
