# Supplementary material for: In-season weather data provide reliable yield estimates of maize and soybean in the US central Corn Belt
Source: Int J Biometeorol. 2020 Nov 21;65(4):489–502. doi: 10.1007/s00484-020-02039-z (PMC7985103; doi:10.1007/s00484-020-02039-z)
Supplement: Supplementary file 1 — (DOCX 11544 kb). [file 484_2020_2039_MOESM1_ESM.docx]

**In-season weather data provide reliable yield estimates of maize and soybean in the US central Corn Belt.**

Vijaya R. Joshi^1,2^, Maciej J. Kazula^1^, Jeffrey A. Coulter^1^, Seth L. Naeve^1^ and Axel Garcia y Garcia^1,2*^

^1^Department of Agronomy and Plant Genetics, University of Minnesota, St. Paul, MN-55108, USA
^2^Southwest Research and Outreach Center, University of Minnesota, Lamberton, MN-56152, USA

^*^Email address: [axel@umn.edu](mailto:axel@umn.edu)

Supplementary materials


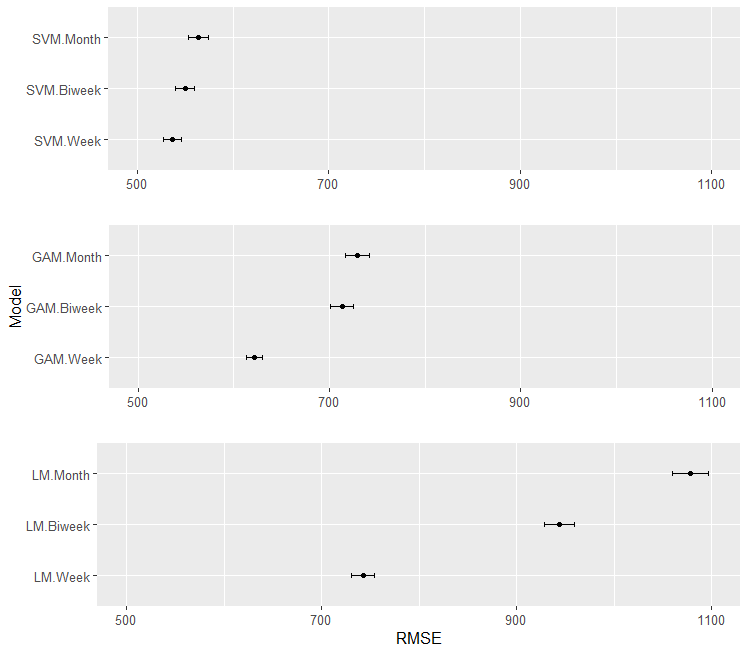


**Fig. S1** Root mean square error (RMSE) of maize yield estimation during model development for the support vector machine (SVM), generalized additive model (GAM), and multiple linear regression (LM) models trained with weather predictors at weekly, biweekly, and monthly time-scales in Iowa.


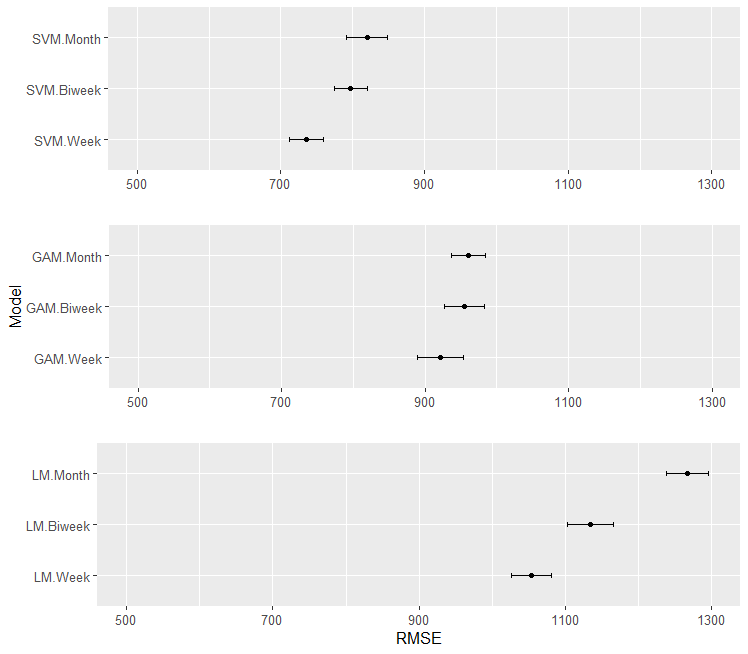


**Fig. S2** Root mean square error (RMSE) of maize yield estimation during model development for the support vector machine (SVM), generalized additive model (GAM), and multiple linear regression (LM) models trained with weather predictors at weekly, biweekly, and monthly time-scales in Illinois.


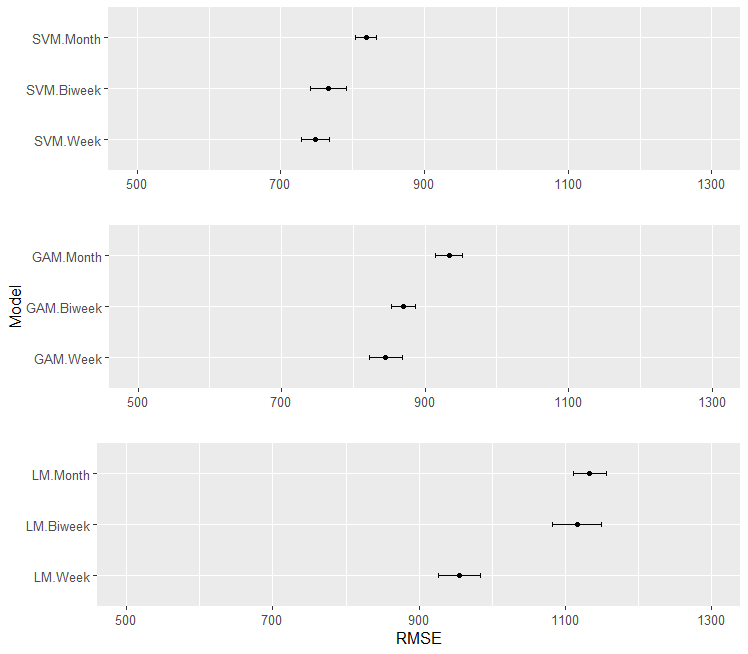


**Fig. S3** Root mean square error (RMSE) of maize yield estimation during model development for the support vector machine (SVM), generalized additive model (GAM), and multiple linear regression (LM) models trained with weather predictors at weekly, biweekly, and monthly time-scales in Indiana.


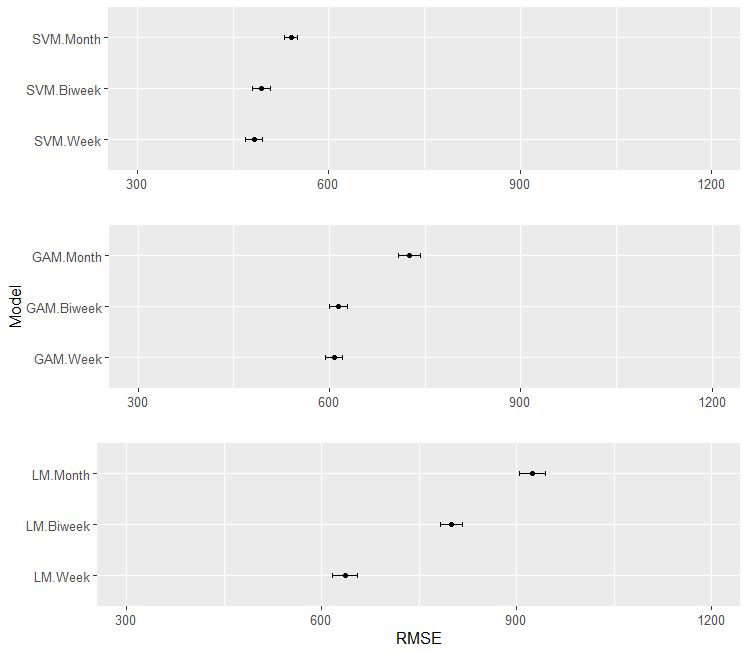


**Fig. S4** Root mean square error (RMSE) of maize yield estimation during model development for the support vector machine (SVM), generalized additive model (GAM), and multiple linear regression (LM) models trained with weather predictors at weekly, biweekly, and monthly time-scales in Minnesota.


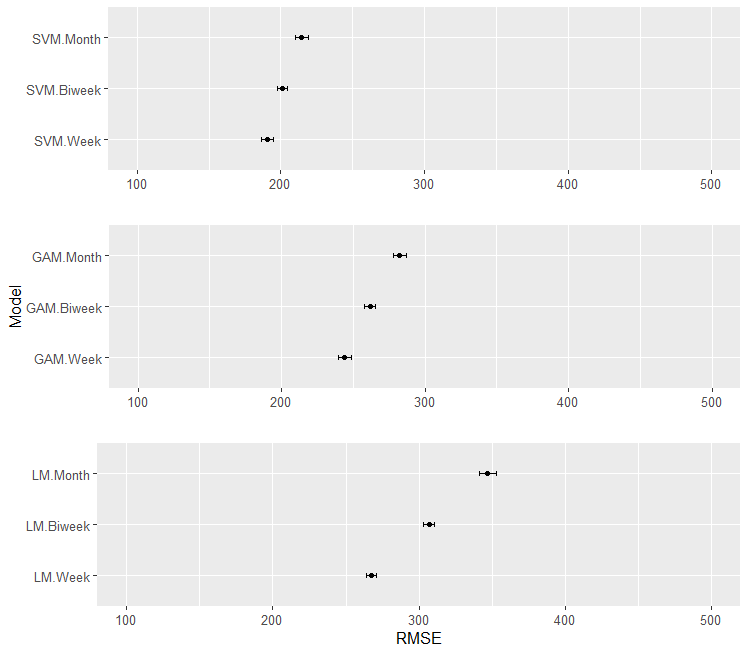


**Fig. S5** Root mean square error (RMSE) of soybean yield estimation during model development for the support vector machine (SVM), generalized additive model (GAM), and multiple linear regression (LM) models trained with weather predictors at weekly, biweekly, and monthly time-scales in Iowa.


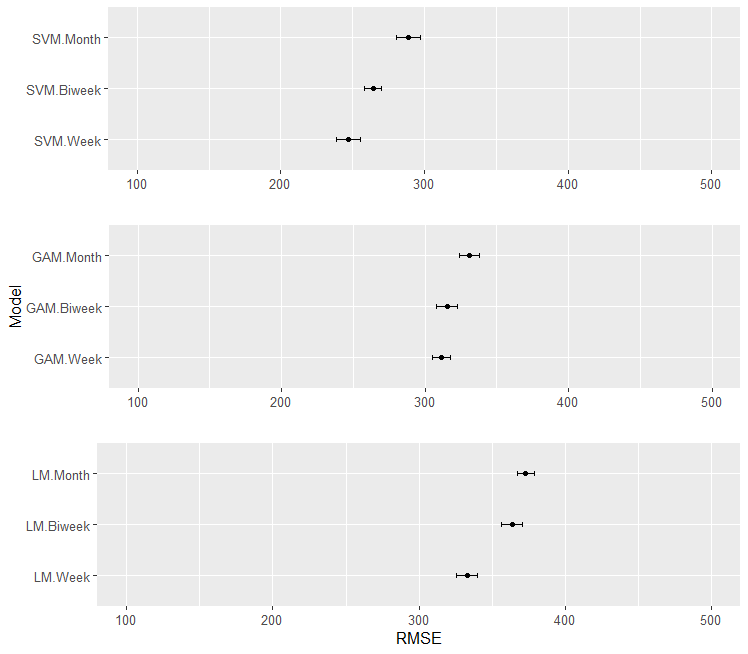


**Fig. S6** Root mean square error (RMSE) of soybean yield estimation during model development for the support vector machine (SVM), generalized additive model (GAM), and multiple linear regression (LM) models trained with weather predictors at weekly, biweekly, and monthly time-scales in Illinois.


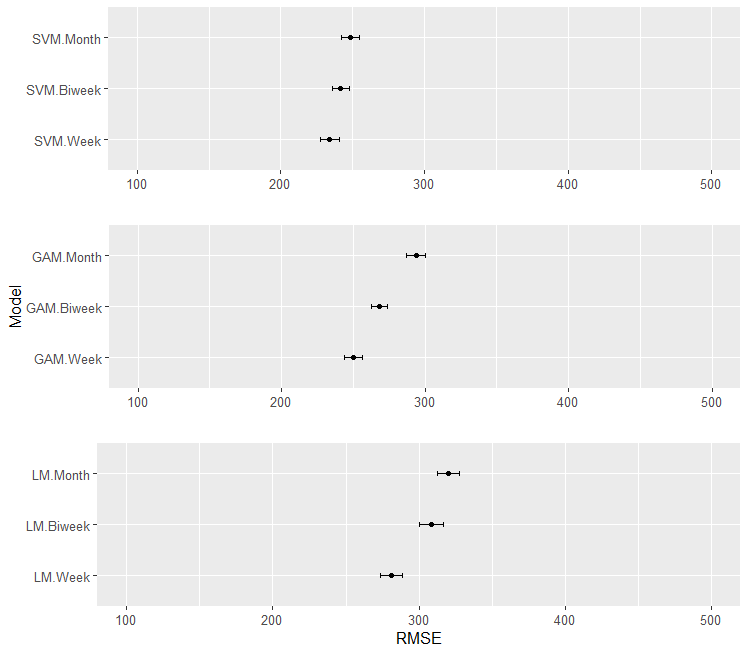


**Fig. S7** Root mean square error (RMSE) of soybean yield estimation during model development for the support vector machine (SVM), generalized additive model (GAM), and multiple linear regression (LM) models trained with weather predictors at weekly, biweekly, and monthly time-scales in Indiana.


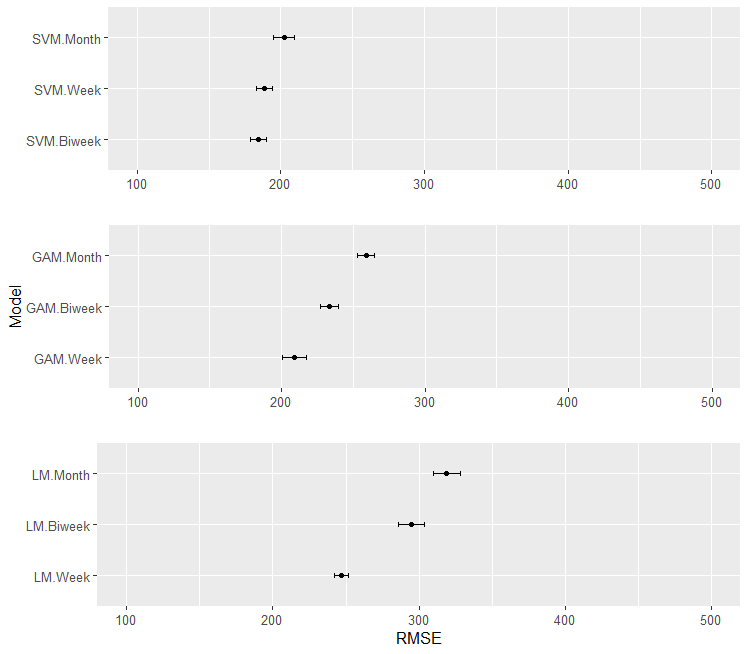


**Fig. S8** Root mean square error (RMSE) of soybean yield estimation during model development for the support vector machine (SVM), generalized additive model (GAM), and multiple linear regression (LM) models trained with weather predictors at weekly, biweekly, and monthly time-scales in Minnesota.


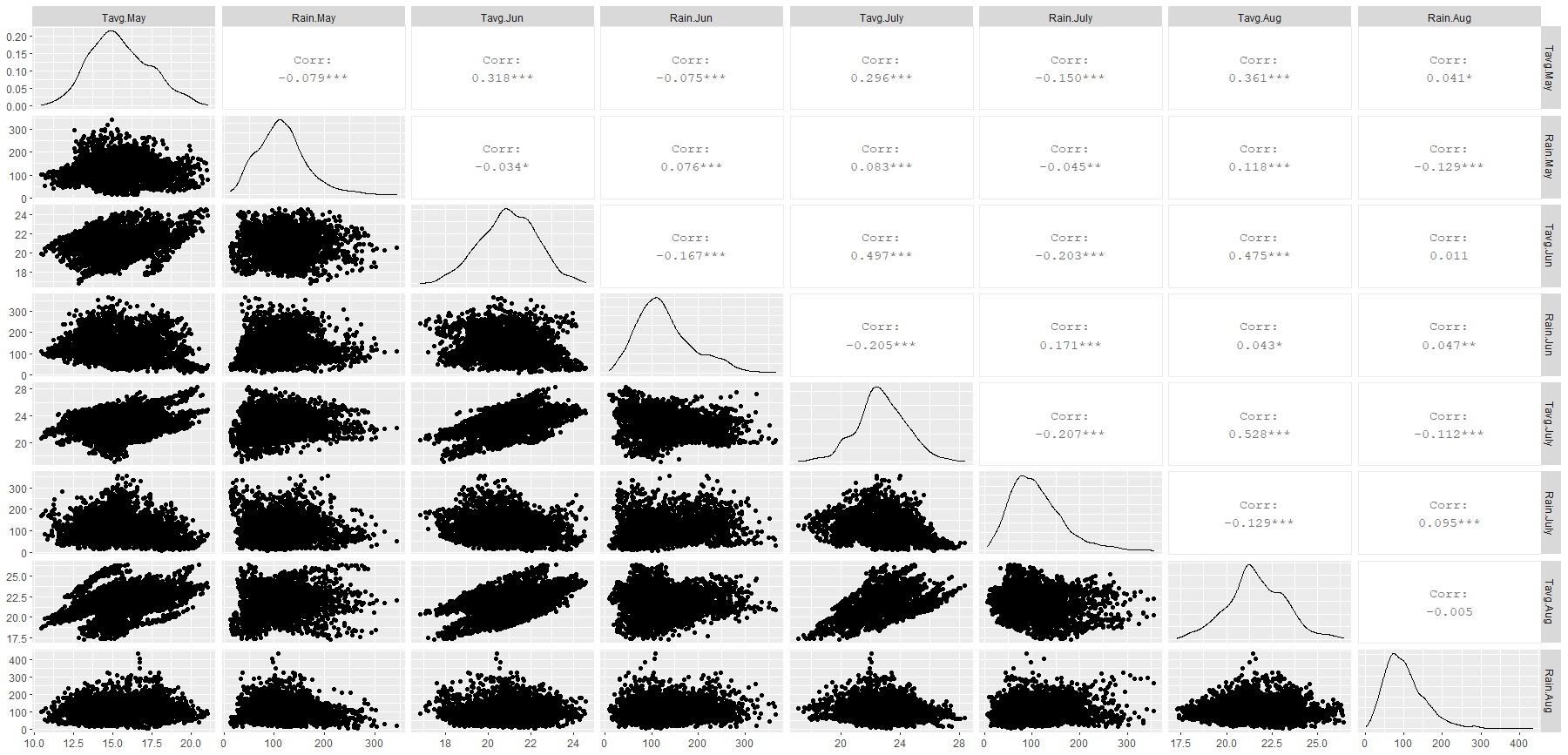


**Fig. S9** Correlation matrix showing scatterplot of each pair of monthly weather variables, weather variable distribution, and Pearson correlation values.
